# Lifestyle factors modified the mediation role of liver fibrosis in the association between occupational physical activity and blood pressure

**DOI:** 10.3389/fpubh.2024.1383065

**Published:** 2024-06-26

**Authors:** Shangyi Zhang, Zhenlong Chen, Xinman Jiang, Shenglan Zhou, Yanru Liu, Mingsheng Liu, Xiayun Dai, Bifeng Lu, Guilin Yi, Wenjun Yin

**Affiliations:** ^1^Wuhan Prevention and Treatment Center for Occupational Diseases (School of Public Health of Joint Training Base for Graduate Students, Hubei University of Medicine), Wuhan, Hubei, China; ^2^School of Public Health, Hubei University of Medicine, Shiyan, Hubei, China

**Keywords:** occupational physical activity, blood pressure, liver fibrosis, lifestyle factor, mediated effect

## Abstract

**Objectives:**

The study aimed to estimate the role of liver fibrosis in the association between occupational physical activity (OPA) and blood pressure (BP), which is modified by lifestyle factors.

**Methods:**

The questionnaire survey and physical examination were completed among 992 construction workers in Wuhan, China. Associations between OPA or lifestyle factors and liver fibrosis indices and blood pressure were assessed using generalized additive models. The mediation analysis was used to evaluate the role of liver fibrosis in the association between OPA and lifestyle factors and BP.

**Results:**

Moderate/high OPA group workers had an increased risk of liver fibrosis [odds ratio (OR) = 1.69, 95% confidence intervals (CI): 1.16–2.47, *P* < 0.05] compared with low OPA group workers. Smoking or drinking alcohol was related to liver fibrosis (aspartate aminotransferase to platelet ratio index: OR = 2.22, 95% CI: 1.07–4.62 or OR = 2.04, 95% CI: 1.00–4.15; *P* < 0.05). Compared with non-drinkers, drinkers were related to a 2.35-mmHg increase in systolic blood pressure (95% CI: 0.09–4.61), and a 1.60-mmHg increase in diastolic blood pressure (95% CI: 0.08–3.13; *P* < 0.05). We found a significant pathway, “OPA → liver fibrosis → blood pressure elevation,” and lifestyle factors played a regulatory role in the pathway.

**Conclusion:**

OPA or lifestyle factors were associated with liver fibrosis indices or BP in construction workers. Furthermore, the association between OPA and BP may be partially mediated by liver fibrosis; lifestyle factors strengthen the relationship between OPA and BP and the mediation role of liver fibrosis in the relationship.

## 1 Introduction

In recent times, the number of people with cardiovascular diseases (CVD) worldwide has reached 520 million, and the number of deaths increased to 18.6 million in 2019 (1). Hypertension is a major risk factor for CVD and is one of the leading causes of premature death worldwide. Approximately 1.28 billion people have hypertension worldwide, of whom 245 million live in China (2). A survey of 37,856 participants from 18 cities in China showed that blue-collar workers (such as construction workers, etc.) were the most high-risk group for hypertension, with a prevalence of nearly 30% (3). Therefore, it may be of great significance to explore the risk factors for hypertension and their impact on the cardiovascular health of blue-collar workers.

In recent times, more and more studies have started to explore the association between occupational physical activity (OPA) and blood pressure (BP) in workers, but the results are controversial. Some studies have demonstrated that higher physical activity was associated with a lower risk of hypertension compared to low physical activity (4, 5). However, a Chinese cohort study (*n* = 9,350) found that high OPA workers had a 1.46 times higher risk of new-onset hypertension compared with low OPA workers (*P* < 0.05) (6). Although they both focus on the association between physical activity and hypertension in Asians, the focus in the above studies is on different aspects. First, the subjects have different occupations and workplaces. Second, there are dissimilar applications of grouping criteria for the OPA in these studies. Moreover, previous studies have provided evidence that lifestyle factors were also significantly associated with increased BP or the risk of hypertension (7, 8). A Canadian study (*n* = 1,177) found that individuals drinking alcohol ≥3 times weekly had approximately double the risk of hypertension compared with non-drinkers (*P* < 0.05) (9). A cross-sectional analysis from China (*n* = 1,248) found that former smokers had a 1.48 times higher risk of hypertension compared with never-smokers (*P* < 0.05) (10). A study conducted on South Asian Americans (*n* = 716) found that the relative risk of hypertension was reduced by 67% with regular intake of fruit, vegetables, and other healthy diet habits compared to people who ate fewer fruits and vegetables (11). It is important to explore the role of lifestyle factors in the association between OPA and BP among blue-collar workers. This research will carry out a mediation or moderation analysis to investigate the association between OPA and blood pressure (BP) among blue-collar workers by considering lifestyle factors.

According to the previous study, behavior, gender, and metabolic factors (obesity, blood lipids, and blood glucose) have been proposed as potential risk factors that can give rise to hypertension. Furthermore, some studies indicated that metabolic factors may play a mediating role in the development of hypertension (12, 13). In addition, epidemiological studies pointed out that metabolic factors may be related to liver fibrosis, such as blood lipids, blood glucose, and uric acid (14). Meanwhile, mechanistic studies found that the associations between liver fibrosis and BP are likely complex and bidirectional (15). On the one hand, liver fibrosis can promote systemic inflammation through damage-associated molecular patterns and altered hepatocyte profiles (16). Additionally, liver fibrosis can increase hepatic diacylglycerol, which activates protein kinase C and decreases insulin signaling, resulting in hepatic insulin resistance (17). Increased levels of inflammation and insulin resistance are two important and possible mechanisms underlying the development of hypertension. On the other hand, hypertension can increase hepatic vascular tone, aggravate the burden on the liver, and lead to architectural disturbances in the liver (including fibrosis, nodule formation, etc.) (18). More and more mechanistic evidence suggests that liver fibrosis may promote the development of hypertension (19). In addition, the early stages of liver fibrosis are usually reversible, which may be a breakthrough point in the prevention of hypertension. However, few epidemiological studies have pointed out that liver fibrosis plays a mediating role in the pathogenesis of hypertension.

Our study aims to explore the mediating effect of liver fibrosis on the association between OPA and BP in the occupational population, which is modified by lifestyle factors. This study incorporates three key lifestyle factors (smoking, drinking alcohol, and dietary habits) as moderators. Five liver fibrosis indices were used as mediators, which were calculated by blood lipid, blood glucose, and aspartate aminotransferase, among others Age, gender, seniority, and body mass index were included as covariates.

## 2 Methods

### 2.1 Study population and design

The study was conducted between June and October 2022 among construction workers in Wuhan, China. A total of 1,388 eligible individuals who were aged ≥18 years and had a seniority of ≥1 year were recruited for our study. Participants with missing data on physical examination (*n* = 186) or questionnaire information (*n* = 124) were excluded. Participants with acute upper respiratory tract infections or immune system diseases (including systemic lupus erythematosus, rheumatoid arthritis, etc.) (*n* = 79) were excluded. We additionally excluded participants with diagnosed cardiovascular diseases other than hypertension and infectious diseases (such as viral hepatitis) (*n* = 7). The remaining 992 participants were included in the cross-sectional study. We used the Monte Carlo power analysis to determine the sample size for our proposed mediation model in the application of indirect effects (20). After fitting the models, we found that statistical power was 70%−90% when the correlation coefficient was in the range of 0.10–0.25 (standard deviation = 0.50) among the independent variable, the mediator, and the dependent variable in our study.

Each participant signed an informed consent after an explanation of the research procedures. The study was approved by the Medical Research Ethics Committee of the Wuhan Prevention and Treatment Center for Occupational Diseases.

### 2.2 Questionnaire survey and physical examination

A total of 992 participants were invited to complete a face-to-face questionnaire. Sociodemographic data, including gender, age, educational attainment, occupational history (such as seniority, jobs, etc.), personal and family medical histories, monthly income, and lifestyle factors (such as smoking, drinking alcohol, bland diet habits, etc.), were collected from the participants. Smoking was defined as smoking ≥1 cigarette per day for more than 6 months; drinking alcohol was defined as drinking alcohol ≥1 time per month for more than 6 months; and a bland diet habit was defined as a habit of showing preference for foods that are gentle on the stomach and easy to digest, such as rice, lean meats, and vegetables (21, 22). According to the occupational health standards in China (23), the definition of OPA, based on the intensity index of physical work (calculated from the working time rate, the average energy metabolic rate of an 8-h workday, the sex-based coefficient of physical work, and the pattern coefficient of physical work), is divided into four grades: low OPA (intensity index ≤ 15), moderate OPA (intensity index >15 and intensity index ≤ 20), high OPA (intensity index >20 and intensity index ≤ 25), and extremely-high OPA (intensity index >25). In this study, we refer to the above occupational health standards in Appendix B. Through the characteristics of the occupational description, 338 workers were divided into low OPA groups (including administrative managers, accountants, chefs, storekeepers, etc.), and 654 workers were divided into moderate/high OPA groups (including painters, porters, masonry workers, etc.). In the questionnaire, we designed some questions (including “What is your job?”, “How do you perceive the level of OPA?”, “How many hours do you usually work 1 day?”, etc.). Before the field investigation, each inquirer was trained to improve his or her interrogation skills. In addition, we obtained the relevant OPA information about the different job descriptions and working durations and found that all the workers' duration of OPA was between 8 and 10 h. Additionally, none of the participants took part in any form of leisure-time physical activity. The two-way validation was performed during the questionnaire entry.

Each participant also finished the physical examination. Body weight and height were measured according to the standardized procedures. Body mass index (BMI) = body weight (kg) ÷ height (m^2^). BP and heart rate (HR) were measured by an automated instrument (Omron-705CP; Omron Corp., Tokyo, Japan) (24). Blood biochemistry, including alanine aminotransferase (ALT), aspartate aminotransferase (AST), total cholesterol, and albumin levels, were measured by the automatic biochemistry analyzer (TBA-FX8, Japan). The maximum reference value of AST was 40 μl. Blood routine indicators, including white blood cell count, platelet count, lymphocyte count, mean platelet volume, red blood cell distribution width, and platelet distribution width, were analyzed by an automatic blood cell analyzer (Mindray BC-6800Plus, Shenzhen, China).

According to the formulae described in detail elsewhere and classified based on the presence or absence of liver fibrosis based on cutoff values (25–27): non-alcoholic fatty liver disease fibrosis score (NFS): liver fibrosis was defined as NFS ≥-1.455; fibrosis index based on the four factors (FIB-4): liver fibrosis for participants aged ≤ 49, 50–59, 60–69, and ≥ 70 years was defined as FIB-4 >1.05, >1.24, >1.88, and >1.95, respectively; aspartate aminotransferase to platelet ratio index (APRI): liver fibrosis was defined as APRI >0.5; aspartate aminotransferase to alanine aminotransferase ratio (AAR): liver fibrosis was defined as AAR >1.5; and red blood cell distribution width to platelet ratio (RPR): liver fibrosis was defined as RPR >0.09 (25–27). Hypertension was defined as systolic blood pressure (SBP) ≥140 mmHg and/or diastolic blood pressure (DBP) ≥90 mmHg. Pre-hypertension was defined as SBP between 120 and 139 mmHg and/or DBP between 80 and 89 mmHg (24).

### 2.3 Statistical analysis

The questionnaire information was double-entered into Epidata 3.1. We used the Student's *t*-test and the chi-square test to analyze and compare the data. A generalized additive model (GAM) with natural spline was used to estimate the effects of OPA or lifestyle factors on liver fibrosis and BP. The natural spline can fit a smooth curve to better explain the non-linear relationship between OPA (low OPA = 0 and moderate/high OPA = 1), smoking (no = 0 and yes = 1), drinking alcohol (no = 0 and yes = 1), bland diet habits (no = 1 and yes = 0), liver fibrosis, and BP (28). We adjusted for age, sex, BMI, seniority, marital status, and average monthly income in the models. Judging from the Akaike information criterion value, we determined the degree of freedom of the smooth function corresponding to the preferred model.

FIB-4, APRI, AAR, and RPR were all log-nature (ln)-transformed considering their right-skewed distribution. The percentage changes in the estimated liver fibrosis were calculated according to the following equation: [exp (β) – 1] × 100%, and the regression coefficient β was obtained from the GAM. The result represented estimated changes and 95% confidence intervals (CI) of BP or HR per one-unit increase in the four liver fibrosis indices. The relationship between OPA and lifestyle factors such as liver fibrosis and hypertension was evaluated by odds ratios (OR). R software version 4.2.2 (packages of mgcv, splines, and ggplot2) and GraphPad Prism 9 (GraphPad Software Inc., San Diego, CA, USA) were used for the analysis procedures and visualization.

We hypothesized that OPA was associated with BP, which could be mediated by liver fibrosis (Supplementary Figure S1). Then, we fitted the two-step linear mixed-effect regression models with random intercepts to quantify the mediating effect of liver fibrosis indices on the association between OPA or lifestyle factors and BP (29).


$$\begin{array}{l} M_{ij}\;=\;\beta_{0}+u_{i}+\beta_{1}X_{1ij}\;+\;.....\;+\beta_{p}X_{pij}+\beta_{exposure}exposure_{ij}+\varepsilon_{ij}\\ \;Y_{ij}=\gamma_{0}+g_{0i}+\gamma_{1}X_{1ij}+\;.....\;+\gamma_{p}X_{pij}\\ \;+\gamma_{exposure}exposure_{ij}+\gamma_{M}M_{ij}+\eta_{ij} \end{array}$$

where *i* represents each participant and *j* represents the clinical visit; M_ij_ corresponds to liver fibrosis indices, and γ_ij_ corresponds to BP. β_exposure_ refers to the OPA in exposure-mediator interactions. γ_M_M_ij_ means liver fibrosis indices in the mediator-outcome interactions. The direct and indirect effects (i.e., mediated effects) were represented as γ_exposure_ and β_exposure_ × γ_m_, respectively. The proportion of the total effect mediated was calculated as the percentage of indirect effect over the sum of direct and indirect effect [i.e., (βexposure × γ_M_)/(β_exposure_ × γ_M_ + γ_exposure_)]. All of the mediation analyses were conducted with the PROCESS macro in Statistical Package for the Social Sciences (SPSS) 25.0 statistical software (V2.16.3, by Andrew F. Hayes). Two-tailed *P-*values of < 0.05 were considered to have statistical significance.

## 3 Results

The baseline values of age (47.3 ± 10.2 years vs. 40.1 ± 13.5 years, *P* < 0.05), seniority (14.3 ± 9.1 years vs. 11.3 ± 8.5 years, *P* < 0.05), SBP (129.93 ± 17.85 mmHg vs. 123.88 ± 17.69 mmHg, *P* < 0.05), DBP (77.28 ± 11.52 mmHg vs. 74.49 ± 11.89 mmHg, *P* < 0.05), NFS (−2.26 ± 1.14 vs. −2.74 ± 1.24, *P* < 0.05), FIB-4 [1.02 (0.77–1.36) vs. 0.77 (0.50–1.10), *P* < 0.05], APRI [0.23 (0.19–0.30) vs. 0.22 (0.17–0.29), *P* < 0.05], and AAR [1.12 (0.91–1.35) vs. 0.97 (0.78–1.29), *P* < 0.05] of the moderate/high OPA group were higher than those of low OPA group (Table 1).

**Table 1:** Characteristics of the study population.

**Category**	**Low OPA (*n* = 338)**	**Moderate/ high OPA (*n* = 654)**	***P*-value**
Age (years, mean ± SD)	40.1 ± 13.5	47.3 ± 10.2	< 0.01^a^
Gender (man/woman, *n*, %)	279/59 (82.5/17.5)	563/91 (86.1/13.9)	0.14^c^
Seniority (years, mean ± SD)	11.3 ± 8.5	14.3 ± 9.1	< 0.01^a^
Body mass index (kg/m^2^, *n*, %)	23.7 ± 3.3	23.7 ± 3.4	0.71^a^
Education (< 9/≥9 years, *n*, %)	116/222 (34.3/65.7)	530/124 (81.0/19.0)	< 0.01^c^
Married (yes/no, *n*, %)	202/136 (59.8/40.2)	516/138 (78.9/21.9)	< 0.01^c^
Smoking (yes/no, *n*, %)	134/204 (39.6/60.4)	269/385 (41.1/58.9)	0.03^c^
Drinking alcohol (yes/no, *n*, %)	98/240 (29.0/71.0)	246/390 (40.4/59.6)	< 0.01^c^
Bland diet habit (yes/no, *n*, %)	79/259 (23.4/76.6)	133/521 (20.3/79.7)	0.03^c^
Average monthly earnings (¥ [yuan], *n*, %)			< 0.01^c^
< 5,000	142 (42.0)	300 (45.9)	
5,000–7,500	107 (31.7)	279 (42.7)	
>7,500	89 (26.3)	75 (11.5)	
Systolic blood pressure (mmHg, mean ± SD)	123.88 ± 17.69	129.93 ± 17.85	< 0.01^a^
Diastolic blood pressure (mmHg, mean ± SD)	74.49 ± 11.89	77.28 ± 11.52	< 0.01^a^
Heart rate (time/min, mean ± SD)	80.37 ± 10.91	78.39 ± 11.30	< 0.01^a^
Alanine aminotransferase (IU/L, median, IQR)	19.50 (13.38, 29.15)	17.70 (13.30, 25.10)	< 0.05^b^
AST (IU/L, median, IQR)	19.40 (16.30, 23.50)	20.20 (16.60, 24.50)	0.07^b^
Albumin (g/L, mean ± SD)	46.40 ± 2.73	45.55 ± 2.55	< 0.01^a^
Fasting plasma glucose (moll/L, median, IQR)	4.82 (4.51, 5.34)	4.88 (4.53, 5.37)	0.48^b^
Total cholesterol (mmol/L, median, IQR)	4.67 (4.17, 5.29)	4.74 (4.32, 5.38)	< 0.05^b^
Triglyceride (mmol/L, median, IQR)	2.31 (1.52, 2.31)	2.31 (1.46, 2.31)	0.09^b^
White blood cell count (10^9^/L, median, IQR)	6.06 (5.25, 7.23)	5.73 (4.88, 6.71)	< 0.01^b^
Platelet count (10^9^/L, median, IQR)	222.52 (193.75, 255.00)	221.00 (190.75, 247.25)	0.17^b^
Lymphocyte count (10^9^/L, median, IQR)	1.96 (1.65, 2.31)	1.96 (1.62, 2.26)	0.44^b^
Red blood cell count (10^12^/L, median, IQR)	4.97 (4.62, 5.23)	4.89 (4.60, 5.14)	< 0.01^b^
Mean platelet volume (fl, median, IQR)	10.30 (9.60, 11.00)	10.10 (9.50, 10.90)	0.14^b^
Platelet distribution width (%, median, IQR)	16.20 (15.90, 16.40)	16.10 (15.90, 16.40)	0.51^b^
Red blood cell distribution width (%, median, IQR)	12.80 (12.40, 13.20)	12.80 (12.50, 13.20)	0.13^b^
NFS (mean ± SD)	−2.74 ± 1.24	−2.26 ± 1.14	< 0.01^a^
FIB-4 (median, IQR)	0.77 (0.50, 1.10)	1.02 (0.77, 1.36)	< 0.01^b^
APRI (median, IQR)	0.22 (0.17, 0.29)	0.23 (0.19, 0.30)	< 0.05^b^
AAR (median, IQR)	0.97 (0.78, 1.29)	1.12 (0.91, 1.35)	< 0.01^b^
RPR (median, IQR)	0.06 (0.05, 0.06)	0.06 (0.05, 0.07)	0.11^b^

OPA, occupational physical activity; IQR, interquartile range; AST, aspartate aminotransferase; NFS, non-alcoholic fatty liver disease fibrosis score; FIB-4, fibrosis index based on the four factors; APRI, aspartate aminotransferase to platelet ratio index; AAR, aspartate aminotransferase to alanine aminotransferase ratio; RPR, red blood cell distribution width to platelet.

^a^Student's t-test was used for comparing the means of the continuous variables between the two groups.

^b^The Mann–Whitney test was used to compare differences in non-normal variable distributions between the two groups.

^c^Chi-square test was used to compare categorical variables between the two groups.

**Figure 1:**
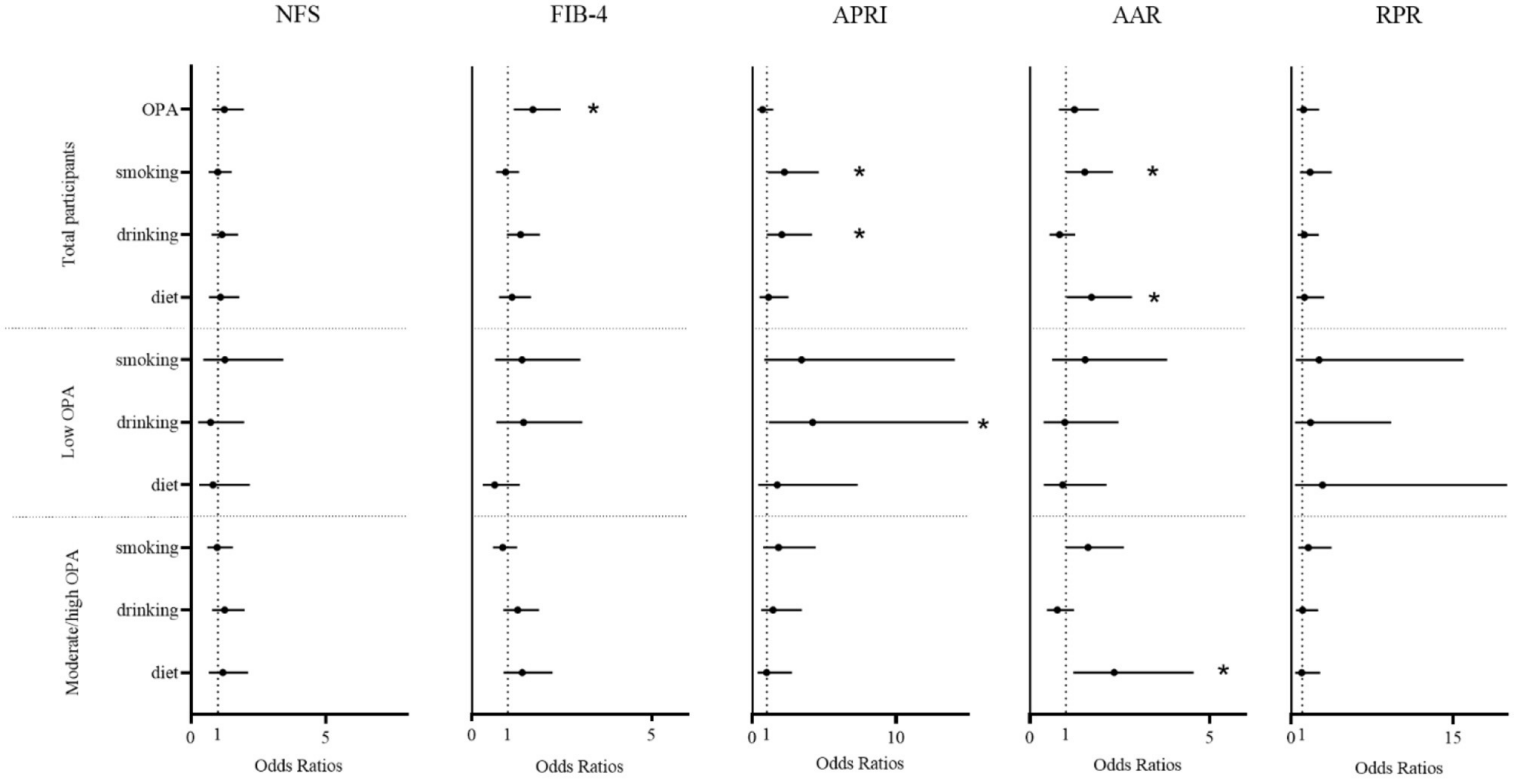
The association between occupational physical activity and lifestyle factors and liver fibrosis. A generalized additive model was used to estimate the odds ratios and 95% confidence intervals, adjusted for age, sex, body mass index, job seniority, marriage, and average monthly earnings. OPA, occupational physical activity; drinking, drinking alcohol; diet, diet habits; NFS, non-alcoholic fatty liver disease fibrosis score; FIB-4, fibrosis index based on the four factors; APRI, aspartate aminotransferase to platelet ratio index; AAR, aspartate aminotransferase to alanine aminotransferase ratio; RPR, red blood cell distribution width to platelet. *P < 0.05.

Figure 1 showed that there were significant positive associations between smoking and liver fibrosis (APRI: OR = 2.22, 95% CI: 1.07–4.62; AAR: OR = 1.52, 95% CI: 1.00–2.31, both *P* < 0.05) and between drinking alcohol and liver fibrosis (APRI: OR = 2.04, 95% CI: 1.00–4.15, *P* < 0.05). Diet habit was associated with liver fibrosis (AAR: OR = 1.71, 95% CI: 1.03–2.84, *P* < 0.05) in all participants, and similar results were found in the moderate/high OPA group (AAR: OR = 2.34, 95% CI: 1.20–4.55, *P* < 0.05). Additionally, we found that the moderate/high OPA group workers had an increased risk of liver fibrosis (FIB-4: OR = 1.69, 95% CI: 1.16–2.47, *P* < 0.05) compared with low OPA group workers. Similar results were found when the liver fibrosis indices were continuous variables (Supplementary Figure S2).

**Figure 2:**
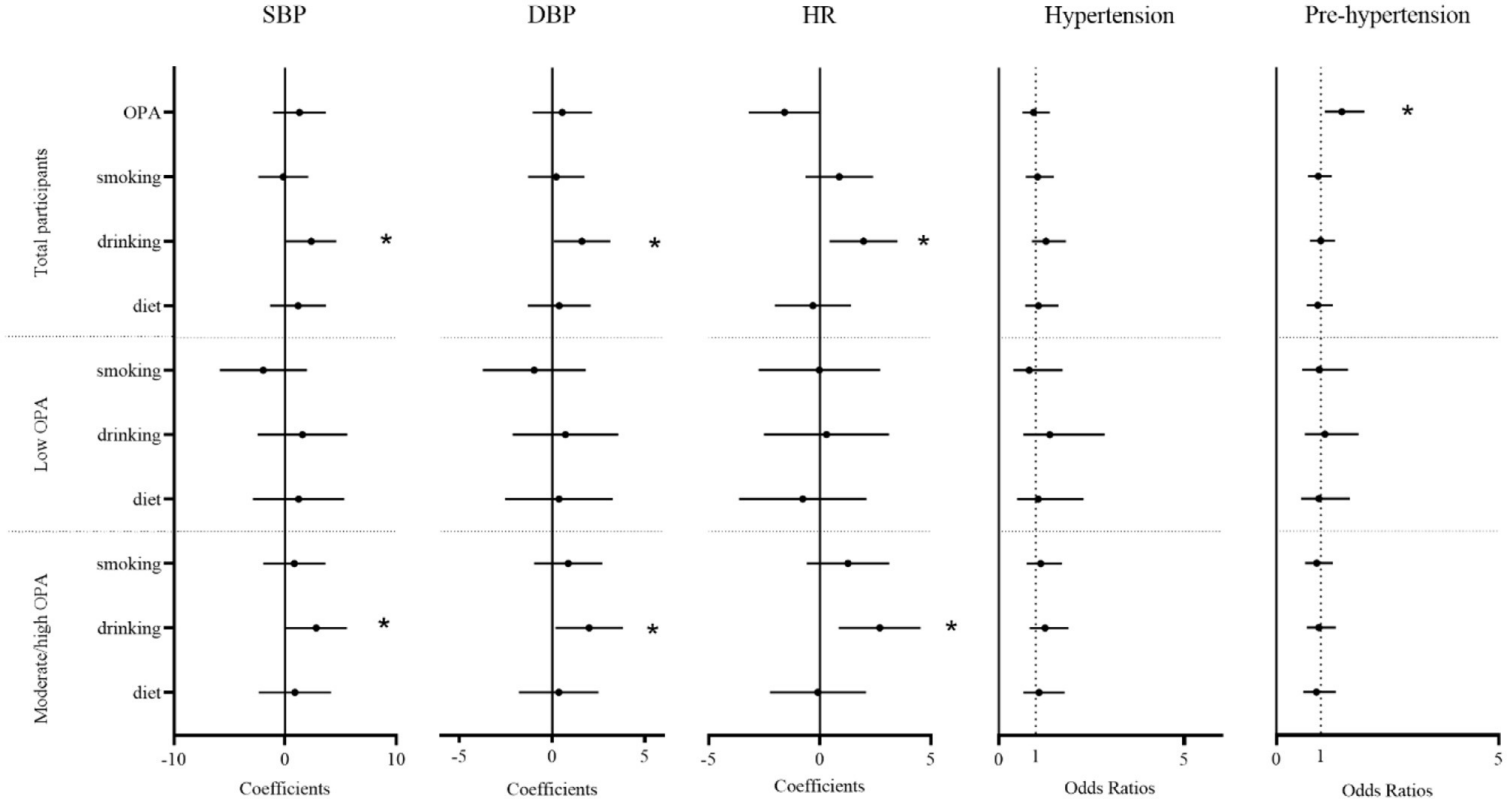
The association between occupational physical activity and lifestyle factor and blood pressure. Estimated changes (95% confidence intervals) in blood pressure, heart rate, and odds ratios (95% confidence intervals) for hypertension and pre-hypertension from different occupational physical activity and lifestyle factors. The result from a generalized additive model adjusted for age, sex, body mass index, job seniority, marriage, and average monthly earnings. OPA, occupational physical activity; drinking, drinking alcohol; diet, diet habits; SBP, systolic blood pressure; DBP, diastolic blood pressure; HR, heart rate. *P < 0.05.

Figure 2 showed that there were significant positive associations between drinking alcohol and BP. In a stratified analysis, the association between drinking alcohol and BP was stronger in the moderate/high OPA group. Among the total number of participants, drinking alcohol was related to a 2.35-mmHg increase in SBP (95% CI: 0.09–4.61), a 1.60-mmHg increase in DBP (95% CI: 0.08–3.13), or a 1.97 time/min increase in HR (95% CI: 0.43–3.50; all *P* < 0.05). In the moderate/high OPA group, drinking alcohol was positively associated with SBP (2.79, 95% CI: 0.02–5.56), DBP (1.99, 95% CI: 0.17–3.82), or HR (2.70, 95% CI: 0.85–4.54; all *P* < 0.05). Additionally, we found that moderate/high OPA group workers had an increased risk of pre-hypertension (OR = 1.47, 95% CI: 1.09–1.98, *P* < 0.05) compared with low OPA group workers.

The mediation analysis study suggests the significance of the pathways “OPA → liver fibrosis → blood pressure elevation.” Liver fibrosis indices (NFS and FIB-4) partially contributed to the effect of OPA on SBP (mediation proportions were 22.56 and 41.39%, both *P* < 0.05), and NFS partially contributed to the effect of OPA on DBP (mediation proportions were 20.33%, *P* < 0.05). However, we did not find evidence of the mediating effect of liver fibrosis indices on the association of OPA and lifestyle factors with HR, hypertension, or pre-hypertension (Supplementary Table S1).

Tables 2–6 show that the mediation analysis study considers lifestyle factors. In Table 2, it is shown that the indirect effect of OPA on SBP is higher in the smoking group than in the non-smoking group (β = 1.25 vs. β = 1.22, both *P* < 0.05). Similarly, the indirect effect of OPA on SBP is higher in the drinking alcohol group than in the non-drinker group (β = 1.62 vs. β = 1.04, both *P* < 0.05). In addition, the indirect effect of OPA on SBP is higher in the no-bland diet group than in the bland diet group (β = 1.24 vs. β = 1.14, both *P* < 0.05). Table 3 shows the significance of the pathways “OPA → liver fibrosis → blood pressure elevation” in the smoking or drinking alcohol group (*P* < 0.05). However, this significance was not observed in the group of non-smokers or non-drinkers of alcohol. As a result, lifestyle factors play a regulatory role in the pathways “OPA → liver fibrosis → blood pressure elevation.”

**Table 2:** The mediating effect of NFS on the association between OPA and blood pressure in different lifestyle groups.

**M**	**W**	**Y**	**Exposure to the mediator (β_exposure_)**	**Mediator to outcome (γ_M_)**	**Mediated effect (Indirect effect, β_exposure_ × γ_M_)**	**Direct effect (γ_exposure_)**	**Mediated proportion (%)**
NFS	Non-smoking	SBP	0.49 (0.31, 0.67)^*^	2.48 (1.12, 3.84)^*^	1.22 (0.50, 2.23)^*^	3.45 (0.35, 6.56)^*^	26.12%
		DBP	0.49 (0.31, 0.67)^*^	0.75 (−0.12, 1.61)	0.37 (−0.03, 0.92)	1.37 (−0.61, 3.35)	–
	Smoking	SBP	0.38 (0.11, 0.65)^*^	3.29 (1.98, 4.61)^*^	1.25 (0.49, 2.45)^*^	5.68 (2.06, 9.30)^*^	18.04%
		DBP	0.38 (0.11, 0.65)^*^	1.58 (0.69, 2.47)^*^	0.61 (0.20, 1.31)^*^	2.60 (0.14, 5.06)^*^	19.00%
	Non-drinking alcohol	SBP	0.46 (0.28, 0.63)^*^	2.28 (1.02, 3.54)^*^	1.04 (0.44, 1.92)^*^	4.01 (1.14, 6.89)^*^	20.59%
		DBP	0.46 (0.28, 0.63)^*^	0.60 (−0.22, 1.41)	0.27 (−0.07, 0.73)	1.62 (−0.24, 3.47)	–
	Drinking alcohol	SBP	0.38 (0.08, 0.67)^*^	3.71 (2.32, 5.10)^*^	1.62 (0.58, 2.96)^*^	4.14 (0.13, 8.15)^*^	28.13%
		DBP	0.38 (0.08, 0.67)^*^	1.80 (0.85, 2.76)^*^	0.68 (0.20, 1.49)^*^	2.50 (−0.21, 5.22)	–
	Bland diet	SBP	0.47 (0.15, 0.78)^*^	2.46 (0.44, 4.47)^*^	1.14 (0.22, 2.81)^*^	5.58 (0.86, 10.30)^*^	16.96%
		DBP	0.47 (0.15, 0.78)^*^	0.72 (−0.62, 2.06)	0.34 (−0.24, 1.40)	2.65 (−0.49, 5.80)	-
	No-bland diet	SBP	0.43 (0.26, 0.60)^*^	2.88 (1.80, 3.96)^*^	1.24 (0.68, 2.06)^*^	4.10 (1.41, 6.79)^*^	23.22%
		DBP	0.43 (0.26, 0.60)^*^	1.19 (0.48, 1.89)^*^	0.51 (0.20, 0.93)^*^	1.85 (0.10, 3.60)^*^	21.61%

OPA, occupational physical activity; drinking, drinking alcohol; NFS, non-alcoholic fatty liver disease fibrosis score.

Mediator M is a non-alcoholic fatty liver disease fibrosis score, Y means the dependent variable, and W represents the moderator. The results were adjusted for age, sex, body mass index, and job seniority.

^*^P < 0.05.

**Table 3:** The mediating effect of FIB-4 on the association between OPA and blood pressure in different lifestyle groups.

**M**	**W**	**Y**	**Exposure to the mediator (β_exposure_)**	**Mediator to outcome (γ_*M*_)**	**Mediated effect (Indirect effect, β_exposure_ × γ_*M*_)**	**Direct effect (γ_exposure_)**	**Mediated proportion (%)**
FIB-4	Non-smoking	SBP	0.30 (0.23, 0.38)^*^	7.39 (4.19, 10.58)^*^	2.25 (1.16, 3.57)^*^	2.43 (−0.74, 5.60)	–
		DBP	0.30 (0.23, 0.38)^*^	1.90 (−0.15, 3.95)	0.58 (−0.03, 1.35)	1.16 (−0.87, 3.18)	–
	Smoking	SBP	0.22 (0.12, 0.33)^*^	10.12 (6.88, 13.35)^*^	2.25 (1.15, 3.68)^*^	4.69 (1.08, 8.29)^*^	32.42%
		DBP	0.22 (0.12, 0.33)^*^	5.86 (3.69, 8.05)^*^	1.30 (0.63, 2.28)^*^	1.90 (−0.54, 4.34)	–
	No-drinking alcohol	SBP	0.31 (0.23, 0.39)^*^	6.96 (4.06, 9.85)^*^	2.15 (1.16, 3.35)^*^	2.90 (−0.04, 5.84)	–
		DBP	0.31 (0.23, 0.39)^*^	2.05 (0.17, 3.93)^*^	0.63 (0.02, 1.32)^*^	1.26 (−0.65, 3.16)	–
	Drinking alcohol	SBP	0.18 (0.06, 0.29)^*^	11.17 (7.47, 14.86)^*^	1.98 (0.73, 3.64)^*^	4.24 (0.22, 8.26)^*^	31.83%
		DBP	0.18 (0.07, 0.29)^*^	6.42 (3.95, 8.89)^*^	1.14 (0.46, 2.17)^*^	2.05 (−0.64, 4.73)	–
	Bland diet	SBP	0.24 (0.10, 0.38)^*^	5.74 (1.19, 10.29)^*^	1.36 (0.39, 3.22)^*^	5.37 (0.62, 10.11)^*^	20.21%
		DBP	0.24 (0.10, 0.38)^*^	1.81 (−1.22, 4.85)	0.43 (−0.29, 1.58)	2.56 (−0.61, 5.72)	–
	No-bland diet	SBP	0.28 (0.21, 0.35)^*^	9.24 (6.59, 11.88)^*^	2.57 (1.65, 3.70)^*^	2.77 (0.06, 5.49)^*^	48.13%
		DBP	0.28 (0.21, 0.35)^*^	4.16 (2.42, 5.89)^*^	1.16 (0.64, 1.83)^*^	1.21 (−0.57, 2.99)	–

OPA, occupational physical activity; FIB-4, fibrosis index based on the four factors.

Mediator M is fibrosis index based on the four factors, Y means the dependent variable, and W represents the moderator. The results were adjusted for age, sex, body mass index, and job seniority.

^*^P < 0.05.

**Table 4:** The mediating effect of APRI on the association between OPA and blood pressure in different lifestyle groups.

**M**	**W**	**Y**	**Exposure to the mediator (β_exposure_)**	**Mediator to outcome (γ_*M*_)**	**Mediated effect (Indirect effect, β_exposure_ × γ_*M*_)**	**Direct effect (γ_exposure_)**	**Mediated proportion (%)**
APRI	Non-smoking	SBP	0.06 (−0.01, 0.12)	1.06 (−2.74, 4.87)	0.06 (−0.12, 0.51)	4.61 (1.53, 7.69)^*^	–
		DBP	0.06 (−0.01, 0.12)	−0.77 (−3.17, 1.63)	−0.04 (−0.29, 0.07)	1.78 (−0.16, 3.72)	–
	Smoking	SBP	0.03 (−0.07, 0.13)	4.31 (0.66, 7.95)^*^	0.12 (−0.28, 0.75)	6.82 (3.15, 10.49)^*^	–
		DBP	0.03 (−0.07, 00.13)	2.69 (0.25, 5.13)^*^	0.07 (−0.20, 0.51)	3.13 (0.67, 5.59)^*^	–
	No-drinking alcohol	SBP	0.07 (0.00, 0.13)^*^	0.90 (−2.58, 4.38)	0.06 (−0.14, 0.47)	4.99 (2.14, 7.85)^*^	–
		DBP	0.07 (0.00, 0.13)^*^	−0.73 (−2.96, 1.50)	−0.05 (−0.30, 0.08)	1.94 (0.11, 3.77)^*^	–
	Drinking alcohol	SBP	−0.02 (−0.13, 0.08)	4.62 (0.54, 8.69)^*^	−0.10 (−0.96, 0.32)	6.32 (2.19, 10.45)^*^	–
		DBP	−0.02 (−0.13, 0.08)	3.13 (0.44, 5.82)^*^	−0.07 (−0.72, 0.19)	3.25 (0.53, 5.98)^*^	–
	Bland diet	SBP	0.05 (−0.07, 0.17)	1.63 (−3.72, 6.99)	0.08 (−0.19, 0.93)	6.64 (1.95, 11.33)^*^	–
		DBP	0.05 (−0.07, 0.17)	−1.44 (−4.97, 2.09)	−0.08 (−0.63, 0.10)	3.06 (−0.03, 6.16)	–
	No-bland diet	SBP	0.04 (−0.03, 0.10)	2.77 (−0.28, 5.82)	0.10 (−0.05, 0.45)	5.24 (2.55, 7.93)^*^	–
		DBP	0.04 (−0.03, 0.10)	1.59 (−0.38, 3.55)	0.06 (−0.03, 0.28)	2.31 (0.57, 4.04)^*^	–

OPA, occupational physical activity; APRI, aspartate aminotransferase to platelet ratio index.

Mediator M is the aspartate aminotransferase to platelet ratio index, Y means the dependent variable, and W represents the moderator. The results were adjusted for age, sex, body mass index, and job seniority.

^*^P < 0.05.

**Table 5:** The mediating effect of AAR on the association between OPA and blood pressure in different lifestyle groups.

**M**	**W**	**Y**	**Exposure to the mediator (β_exposure_)**	**Mediator to outcome (γ_*M*_)**	**Mediated effect (Indirect effect, β_exposure_ × γ_*M*_)**	**Direct effect (γ_exposure_)**	**Mediated proportion (%)**
AAR	Non-smoking	SBP	0.11 (0.06, 0.17)^*^	−3.03 (−7.51, 1.44)	−0.34 (−0.98, 0.08)	5.01 (1.91, 8.12)^*^	–
		DBP	0.11 (0.06, 0.17)^*^	−4.60 (−7.41, −1.80)	−0.52 (−1.04, −0.18)	2.25 (0.30, 4.20)^*^	–
	Smoking	SBP	0.15 (0.07, 0.22)^*^	4.55 (−0.22, 9.33)	0.66 (0.06, 1.72)^*^	6.27 (2.53, 10.02)^*^	–
		DBP	0.15 (0.07, 0.22)^*^	4.71 (1.53, 7.89)^*^	0.69 (0.21, 1.51)^*^	2.52 (0.03, 5.01)^*^	21.50%
	No-drinking alcohol	SBP	0.12 (0.06, 0.17)^*^	−2.11 (−6.35, 2.12)	−0.25 (−0.83, 0.17)	5.30 (2.41, 8.19)^*^	–
		DBP	0.12 (0.06, 0.17)^*^	−3.02 (−5.72, −0.32)	−0.35 (−0.83, −0.07)	2.24 (0.40, 4.09)^*^	–
	Drinking alcohol	SBP	0.15 (0.06, 0.23)^*^	4.05 (−1.10, 9.19)	0.60 (−0.04, 1.62)	5.62 (1.41, 9.83)^*^	–
		DBP	0.15 (0.06, 0.23)^*^	3.30 (−0.09, 6.69)	0.49 (0.05, 1.22)^*^	2.69 (−0.08, 5.47)	–
	Bland diet	SBP	0.10 (0.01, 0.18)^*^	−4.47 (−12.06, 3.11)	−0.44 (−1.56, 0.14)	7.17 (2.43, 11.90)^*^	–
		DBP	0.10 (0.01, 0.18)^*^	−0.58 (−5.60, 4.43)	−0.06 (−0.64, 0.52)	3.05 (−0.09, 6.18)	–
	No-bland diet	SBP	0.14 (0.09, 0.20)^*^	0.98 (−2.68, 4.64)	0.14 (−0.31, 0.65)	5.20 (2.46, 7.95)^*^	–
		DBP	0.14 (0.09, 0.20)^*^	−0.63 (−2.99, 1.73)	−0.09 (−0.46, 0.26)	2.45 (0.69, 4.22)^*^	–

OPA, occupational physical activity; AAR, aspartate aminotransferase to alanine aminotransferase ratio.

Mediator M is the aspartate aminotransferase to alanine aminotransferase ratio, Y means the dependent variable, and W represents the moderator. The results were adjusted for age, sex, body mass index, and job seniority.

^*^P < 0.05.

**Table 6:** The mediating effect of RPR on the association between OPA and blood pressure in different lifestyle groups.

**M**	**W**	**Y**	**Exposure to the mediator (β_exposure_)**	**Mediator to outcome (γ_*M*_)**	**Mediated effect (Indirect effect, β_exposure_ × γ_*M*_)**	**Direct effect (γ_exposure_)**	**Mediated proportion (%)**
RPR	Non-smoking	SBP	0.03 (−0.01, 0.07)	−2.32 (−8.91, 4.28)	−0.07 (−0.46, 0.10)	4.75 (1.67, 7.82)	–
		DBP	0.03 (−0.01, 0.07)	−1.91 (−6.07, 2.25)	−0.06 (−0.32, 0.05)	1.79 (−0.15, 3.73)	–
	Smoking	SBP	0.01 (−0.04, 0.06)	6.92 (−0.07, 13.91)	0.09 (−0.27, 0.66)	6.85 (3.17, 10.53)^*^	–
		DBP	0.01 (−0.04, 0.06)	3.74 (−0.94, 8.43)	0.05 (−0.12, 0.42)	3.15 (0.69, 5.62)^*^	–
	No-drinking alcohol	SBP	0.03 (−0.01, 0.07)	−1.55 (−7.73, 4.63)	−0.05 (−0.38, 0.12)	5.10 (2.25, 7.95)^*^	–
		DBP	0.03 (−0.01, 0.07)	−1.17 (−5.13, 2.78)	−0.04 (−0.25, 0.07)	1.92 (0.10, 3.75)^*^	–
	Drinking alcohol	SBP	0.00 (−0.05, 0.06)	6.96 (−0.61, 14.53)	0.02 (−0.50, 0.57)	6.20 (2.06, 10.34)^*^	–
		DBP	0.00 (−0.05, 0.06)	3.26 (−1.75, 8.26)	0.01 (−0.21, 0.36)	3.17 (0.44, 5.91)^*^	–
	Bland diet	SBP	0.06 (−0.01, 0.12)	−0.03 (−9.93, 9.87)	−0.002 (−0.71, 0.68)	6.73 (2.00, 11.45)^*^	–
		DBP	0.06 (−0.01, 0.12)	−1.48 (−8.00, 5.05)	−0.09 (−0.71, 0.26)	3.07 (−0.04, 6.19)	–
	No-bland diet	SBP	0.01 (−0.03, 0.04)	1.86 (−3.637.36)	0.02 (−0.06, 0.28)	5.33 (2.64, 8.02)^*^	–
		DBP	0.01 (−0.03, 0.04)	0.99 (−2.56, 4.54)	0.01 (−0.04, 0.17)	2.36 (0.62, 4.09)^*^	–

OPA, occupational physical activity; RPR, red blood cell distribution width to the platelet.

Mediator M is red blood cell distribution width to platelet, Y means dependent variable, and W represents the moderator. The results were adjusted for age, sex, body mass index, and job seniority.

^*^P < 0.05.

## 4 Discussion

We observed a significant positive association between OPA and the risk of liver fibrosis, and OPA strengthened the association between lifestyle factors and liver fibrosis indices as well. A cross-sectional study (*n* = 167,000) conducted in the north of the Netherlands found that OPA was positively associated with the liver fibrosis index (*P* < 0.05) (30). This finding is consistent with the results of our study. Mechanistic studies have shown that high-intensity physical activity results in micro-tears or damage in the muscle fibers and leads to increased inflammatory cytokine (such as interleukin-8, interleukin-10, C-reactive protein, etc.) levels (31, 32). Not having enough time to recover after intense physical activity may lead to persistent inflammation (31), which is the prerequisite for the formation of liver fibrosis and the driving force behind its progression (33). However, a prospective study of Americans (*n* = 755,459) found that engaging in leisure-time physical activity was associated with a significantly lower risk of liver cancer compared with engaging in no physical activity (*P* < 0.05) (34). For the most part, OPA is not equal to leisure-time physical activity and may even be harmful to our health (35). First, the purpose of the two physical activities is different. Leisure-time physical activity refers to planned, repetitive activities (such as swimming and running) to maintain physical fitness (36); OPA refers to a variety of activities arising from work, including standing for long periods of time, repetitive bending over, and pushing and pulling (37). Second, the oxygen consumption is different between the two physical activities. Leisure-time physical activity generally involves shorter periods of moderate-intensity aerobic exercise, while OPA usually involves a large amount of anaerobic exercise (such as repetitive work and prolonged static posture tasks), which is often more than 40 h/week and lacks sufficient recovery time (38, 39).

Our results showed a significant positive association between lifestyle factors and liver fibrosis. A cohort study of Koreans (*n* = 1,070,991) found that men who smoked ≥20 pack-years had 1.29 times (95% CI: 1.18–1.42) higher incidence of liver fibrosis compared to non-smokers; women who smoked ≥10 pack-years had 1.75 times (95% CI: 1.12–2.73) increased risk of liver fibrosis compared to non-smokers (*P* < 0.05) (40). The results suggest that smokers have a higher risk of liver fibrosis than non-smokers. Unhealthy diet habits also play an important role in liver fibrosis. A toxicological study using male KK-Ay/TaJcl mice showed that their liver tissue sections showed an increase in the total area of hepatic fibrosis by 2% after feeding the diet containing 40 kcal% fat, 20 kcal% fructose, 2% cholesterol, and 0.5% cholic acid for 12 weeks (41). Some mechanistic studies found that an excessive high-fat diet can lead to hepatocyte injury and death through altered lipid and glucose metabolism or inflammation. Furthermore, it can induce activation of hepatic stellate cells and collagen deposition and ultimately result in liver fibrosis and even hepatocellular cancer (42, 43).

Our study found that OPA was positively associated with BP. A longitudinal study in Copenhagen (*n* = 104,046) found a 35% increased risk of major adverse cardiovascular events (e.g., cardiovascular death, cardiac arrest, cardiogenic shock, etc.) in the high OPA group compared to the low OPA group (44). Mechanistic studies suggest that this increase may be due to sympathetic hyperactivity as a result of high OPA and the excessive secretion of norepinephrine results in higher BP (45). In addition, studies have found that high OPA-workers have long working hours and short rest periods, which lead to fatigue, increased systemic inflammation, and elevated BP (46). Our study found a positive association between OPA and pre-hypertension, which can be considered as an indicator of hypertension risk in blue-collar workers.

Our study demonstrated that drinking alcohol was positively associated with BP, and the results were stronger in the moderate/high OPA group. A cohort study (*n* = 599,912) from 19 countries in Europe showed that drinking alcohol was positively associated with the risk of hypertension (OR = 1.24, 95% CI: 1.15–1.33, *P* < 0.05) (47). A meta-analysis of drinking alcohol and blood pressure found that the average SBP was 4.90 mmHg higher for those consuming 48 g of alcohol daily compared with no-drinking alcohol (48). The association between drinking alcohol and BP could be modified by other factors such as sex and occupation (48). This finding is consistent with our results. Mechanistic studies have shown that drinking alcohol can change the release of acetylcholine to activate sympathetic nerves and increase vascular resistance, thereby increasing the contractility of the heart muscle and leading to increased BP (49).

Our study found that liver fibrosis was positively associated with BP. This finding indicates that the liver fibrosis index contributes to the prediction of hypertension. A Framingham Heart Study (*n* = 3,276) found that liver fibrosis was positively associated with hypertension (OR = 1.52, *P* < 0.05) (50). A study of 4,164 Chinese hypertensive patients also found a positive association between liver fibrosis and cardiovascular disease (hazard ratios = 3.13) (*P* < 0.05) (51). A comprehensive review written by Eric P et al. summarizes some mechanisms of liver fibrosis and its role in increased BP, including oxidative stress and lipid metabolism (52). Moreover, increasing evidence indicates that liver fibrosis can worsen insulin resistance and release multiple pro-inflammatory, vasoactive, and pro-thrombotic factors. This finding may increase the likelihood of developing hypertension and other cardiovascular diseases (53).

Meanwhile, the mediation analysis suggests that liver fibrosis indices could partially mediate the association of OPA with BP, and lifestyle factors may be regarded as moderators. Mechanistic studies found that inflammation may play an important role in this pathway. After an inflammatory response in the body caused by external stimuli, liver injury can be exacerbated by triggering the activation of hepatic stellate cells, which can transform into fibroblasts and promote liver fibrosis by secreting type I collagen (42, 54). Liver fibrosis and the development of portal hypertension generate an increase in intrahepatic blood flow resistance, which puts strain on the cardiovascular system and increases the risk of CVD (55). In addition, our study found that lifestyle factors moderated the mediating effect of liver fibrosis on the relationship between OPA and BP. A perspective study (*n* = 504,009) conducted in China found that participants with 2–4 healthy lifestyle factors (such as smoking, drinking alcohol, and physical activity, etc.) had 12%−44% lower risks of severe liver disease, compared with those with 0–1 healthy lifestyle factor (56). At present, most studies agree that physical activity is beneficial for liver health. Our results suggest that it is important to regulate the intensity and duration of physical activity properly (57). Moreover, a cross-sectional study (*n* = 2,189) conducted in China found that people with high physical activity who eat < 1 egg/day were associated with a higher risk of hypertension compared with those with moderate physical activity who only eat 1 egg/day (OR = 2.9, *P* < 0.05) (58). This finding is consistent with our results: to reduce the incidence of hypertension, both appropriate OPA and healthy lifestyle factors are indispensable.

Taken together, OPA and unhealthy lifestyle factors were positively associated with liver fibrosis, or BP, respectively. Liver fibrosis can be used as a predictor for hypertension, providing new insights into prevention. In moderate- and high-OPA group workers, unhealthy lifestyle factors can lead to increased BP and increased cardiovascular burden. A healthy lifestyle factor and reasonable arrangements of rest breaks for different OPA are beneficial to chronic disease management in construction workers.

Our study is the first to examine the effect of behavioral factors on hypertension in Chinese blue-collar workers, which may be partially mediated by liver fibrosis. The study also contains several limitations. First, we used a cross-section study for data analysis, and the strength of the causal relationships was insufficient. In the future, we will follow up annually and analyze their longitudinal data. Second, due to the limitation of the sample size, our findings should be interpreted with caution and need to be validated with a larger sample size. Third, we did not evaluate the metabolic equivalent of the task but grouped it according to occupation and self-reported OPA, which may have some information bias. In addition, liver fibrosis levels were obtained using a formula and therefore have the potential to overestimate or underestimate the value of liver fibrosis compared to direct measures. Finally, our observational study design cannot determine the underlying mechanisms and temporality of the relationships, which may need to be explored by more mechanistic studies.

## Data availability statement

The raw data supporting the conclusions of this article will be made available by the authors, without undue reservation.

## Ethics statement

The studies involving humans were approved by Medical Research Ethics Committee of Wuhan Prevention and Treatment Center for Occupational Diseases. The studies were conducted in accordance with the local legislation and institutional requirements. The participants provided their written informed consent to participate in this study.

## Author contributions

SZha: Data curation, Methodology, Software, Visualization, Writing—original draft, Writing—review & editing. ZC: Conceptualization, Formal analysis, Supervision, Writing- review & editing. XJ: Conceptualization, Investigation, Writing—original draft. SZho: Conceptualization, Investigation, Supervision, Writing—review & editing. YL: Conceptualization, Investigation, Visualization, Writing—review & editing. ML: Formal analysis, Investigation, Writing—review & editing. XD: Investigation, Methodology, Writing—review & editing. BL: Investigation, Methodology, Writing—review & editing. GY: Conceptualization, Investigation, Supervision, Writing—review & editing. WY: Conceptualization, Funding acquisition, Investigation, Methodology, Resources, Software, Writing—original draft, Writing—review & editing.

## References

[1] Roth GA Mensah GA Johnson CO Addolorato G Ammirati E Baddour LM. Global burden of cardiovascular diseases and risk factors, 1990-2019: update from the GBD 2019 study. J Am Coll Cardiol. (2020) 76:2982–3021. 10.1016/j.jacc.2020.11.010 33309175 PMC7755038

[2] NCD Risk Factor Collaboration (NCD-RisC). Worldwide trends in hypertension prevalence and progress in treatment and control from 1990 to 2019: a pooled analysis of 1201 population-representative studies with 104 million participants. Lancet. (2021) 398:957–80. 10.1016/S0140-6736(21)01330-1 34450083 PMC8446938

[3] Shen Y Wang X Wang Z Zhang L Chen Z Zhu M. Prevalence, awareness, treatment, and control of hypertension among Chinese working population: results of a workplace-based study. J Am Soc Hypertens. (2018) 12:311–22.e2. 10.1016/j.jash.2018.01.013 29483001

[4] Do AD Pham TTP Nguyen CQ Van Hoang D Fukunaga A Yamamoto S. Different associations of occupational and leisure-time physical activity with the prevalence of hypertension among middle-aged community dwellers in rural Khánh Hòa, Vietnam. BMC Public Health. (2023) 23:713. 10.1186/s12889-023-15631-w 37076854 PMC10116664

[5] Guan T Cao M Zheng C Zhou H Wang X Chen Z. Dose-response association between physical activity and blood pressure among Chinese adults: a nationwide cross-sectional study. J Hypertens. (2024) 42:360–70. 10.1097/HJH.0000000000003587 38037282 PMC10763713

[6] Li Q Li R Zhang S Zhang Y He P Zhang Z. Occupational physical activity and new-onset hypertension: a nationwide cohort study in China. Hypertension. (2021) 78:220–9. 10.1161/HYPERTENSIONAHA.121.17281 34058853

[7] Zhang S Qian ZM Chen L Zhao X Cai M Wang C et al. Exposure to air pollution during pre-hypertension and subsequent hypertension, cardiovascular disease, and death: a trajectory analysis of the UK Biobank Cohort. Environ Health Perspect. (2023) 131:17008. 10.1289/EHP10967 36696106 PMC9875843

[8] Beilin LJ. Lifestyle and hypertension–an overview. Clin Exp Hypertens. (1999) 21:749–62. 10.3109/10641969909061005 10423098

[9] Allaire J Lévesque B Poirier P Gagnon C Auclair G Lemire M. Prevalence and determinants of hypertension in the adult Inuit population of Nunavik (Northern Quebec, Canada). Can J Public Health. (2023) 115(Suppl 1):168–79. 10.17269/s41997-023-00774-5 37155001 PMC10830977

[10] Li G Wang H Wang K Wang W Dong F Qian Y. The association between smoking and blood pressure in men: a cross-sectional study. BMC Public Health. (2017) 17:797. 10.1186/s12889-017-4802-x 29017534 PMC5634904

[11] Hussain BM Deierlein AL Kanaya AM Talegawkar SA O'Connor JA Gadgil MD. Concordance between dash diet and hypertension: results from the mediators of atherosclerosis in South Asians Living in America (MASALA) Study. Nutrients. (2023) 15:3611. 10.3390/nu15163611 37630801 PMC10458588

[12] Hidayat MM Agustiningsih D Sabirin RM Wibowo RA. The mediation role of physical fitness in association between muscle-strengthening physical activities and its component with blood pressure among young adults: considering gender and abnormal blood pressure as moderators, moderate-vigorous physical activity, sleep behavior, sedentary behavior, mental wellbeing and BMI as covariates. Front Cardiovas Med. (2023) 10:1158893. 10.3389/fcvm.2023.1158893 37799780 PMC10548210

[13] Cao Z Cheng Y Li S Yang H Sun L Gao Y. Mediation of the effect of serum uric acid on the risk of developing hypertension: a population-based cohort study. J Transl Med. (2019) 17:202. 10.1186/s12967-019-1953-9 31215428 PMC6582569

[14] Xue Y Xu J Li M Gao Y. Potential screening indicators for early diagnosis of NAFLD/MAFLD and liver fibrosis: triglyceride glucose index-related parameters. Front Endocrinol. (2022) 13:951689. 10.3389/fendo.2022.951689 36120429 PMC9478620

[15] Rockey DC Bell PD Hill JA. Fibrosis–a common pathway to organ injury and failure. N Engl J Med. (2015) 372:1138–49. 10.1056/NEJMra1300575 25785971

[16] Song Q Ling Q Fan L Deng Y Gao Q Yang R. Severity of non-alcoholic fatty liver disease is a risk factor for developing hypertension from prehypertension. Chin Med J. (2023) 136:1591–7. 10.1097/CM9.0000000000002111 37027402 PMC10325755

[17] Fujii H Kawada N Japan Japan Study Group of Nafld J-N. The role of insulin resistance and diabetes in nonalcoholic fatty liver disease. Int J Mol Sci. (2020) 21:3863. 10.3390/ijms21113863 32485838 PMC7312931

[18] García-Pagán JC Gracia-Sancho J Bosch J. Functional aspects on the pathophysiology of portal hypertension in cirrhosis. J Hepatol. (2012) 57:458–61. 10.1016/j.jhep.2012.03.007 22504334

[19] Lonardo A Nascimbeni F Mantovani A Targher G. Hypertension, diabetes, atherosclerosis and NASH: cause or consequence? J Hepatol. (2018) 68:335–52. 10.1016/j.jhep.2017.09.021 29122390

[20] Schoemann AM Boulton AJ Short SD. Determining power and sample size for simple and complex mediation models. Soc Psychol Personal Sci. (2017) 8:379–86. 10.1177/1948550617715068

[21] Yang L Zhou Y Sun H Lai H Liu C Yan K. Dose-response relationship between polycyclic aromatic hydrocarbon metabolites and risk of diabetes in the general Chinese population. Environ Pollut. (2014) 195:24–30. 10.1016/j.envpol.2014.08.012 25194268

[22] Weir SBS Akhondi H. Bland Diet. StatPearls. Treasure Island, FL: StatPearls Publishing LLC (2022). 30844169

[23] Ministry of Health of the People's Republic of China. GBZ 2.2-2007: Occupational Exposure Limits for Hazardous Agents in the Workplace Part2: Physical Agents. Beijing: China Standards Press (2007).

[24] Pickering TG Hall JE Appel LJ Falkner BE Graves J Hill MN. Recommendations for blood pressure measurement in humans and experimental animals: part 1: blood pressure measurement in humans: a statement for professionals from the Subcommittee of Professional and Public Education of the American Heart Association Council on High Blood Pressure Research. Circulation. (2005) 111:697–716. 10.1161/01.CIR.0000154900.76284.F6 15699287

[25] Ishiba H Sumida Y Tanaka S Yoneda M Hyogo H Ono M. The novel cutoff points for the FIB4 index categorized by age increase the diagnostic accuracy in NAFLD: a multi-center study. J Gastroenterol. (2018) 53:1216–24. 10.1007/s00535-018-1474-y 29744597

[26] Kim WR Berg T Asselah T Flisiak R Fung S Gordon SC. Evaluation of APRI and FIB-4 scoring systems for non-invasive assessment of hepatic fibrosis in chronic hepatitis B patients. J Hepatol. (2016) 64:773–80. 10.1016/j.jhep.2015.11.012 26626497

[27] Wu X Cai B Su Z Li Y Xu J Deng R. Aspartate transaminase to platelet ratio index and gamma-glutamyl transpeptidase-to-platelet ratio outweigh fibrosis index based on four factors and red cell distribution width-platelet ratio in diagnosing liver fibrosis and inflammation in chronic hepatitis B. J Clin Lab Anal. (2018) 32:e22341. 10.1002/jcla.22341 29251384 PMC6816941

[28] Hastie T Tibshirani R. Generalized additive models for medical research. Stat Methods Med Res. (1995) 4:187–96. 10.1177/096228029500400302 8548102

[29] Bauer DJ Preacher KJ Gil KM. Conceptualizing and testing random indirect effects and moderated mediation in multilevel models: new procedures and recommendations. Psychol Methods. (2006) 11:142–63. 10.1037/1082-989X.11.2.142 16784335

[30] Byambasukh O Zelle D Corpeleijn E. Physical activity, fatty liver, and glucose metabolism over the life course: the lifelines cohort. Am J Gastroenterol. (2019) 114:907–15. 10.14309/ajg.0000000000000168 30865013

[31] Cerqueira É Marinho DA Neiva HP Lourenço O. Inflammatory effects of high and moderate intensity exercise-a systematic review. Front Physiol. (2019) 10:1550. 10.3389/fphys.2019.01550 31992987 PMC6962351

[32] Saidi K Abderrahman AB Hackney AC Bideau B Zouita S Granacher U. Hematology, hormones, inflammation, and muscle damage in elite and professional soccer players: a systematic review with implications for exercise. Sports Med. (2021) 51:2607–27. 10.1007/s40279-021-01522-w 34347283

[33] Calvente CJ Tameda M Johnson CD Del Pilar H Lin YC Adronikou N. Neutrophils contribute to spontaneous resolution of liver inflammation and fibrosis via microRNA-223. J Clin Invest. (2019) 129:4091–109. 10.1172/JCI122258 31295147 PMC6763256

[34] Matthews CE Moore SC Arem H Cook MB Trabert B Håkansson N. Amount and intensity of leisure-time physical activity and lower cancer risk. J Clin Oncol. (2020) 38:686–97. 10.1200/JCO.19.02407 31877085 PMC7048166

[35] Pearce M Strain T Wijndaele K Sharp SJ Mok A Brage S. Is occupational physical activity associated with mortality in UK Biobank? Int J Behav Nutr Phys Act. (2021) 18:102. 10.1186/s12966-021-01154-3 34315448 PMC8314512

[36] Shiri R Falah-Hassani K. Does leisure time physical activity protect against low back pain? Systematic review and meta-analysis of 36 prospective cohort studies. Br J Sports Med. (2017) 51:1410–8. 10.1136/bjsports-2016-097352 28615218

[37] Holtermann A Krause N van der Beek AJ Straker L. The physical activity paradox: six reasons why occupational physical activity (OPA) does not confer the cardiovascular health benefits that leisure time physical activity does. Br J Sports Med. (2018) 52:149–50. 10.1136/bjsports-2017-097965 28798040

[38] Coenen P Huysmans MA Holtermann A Krause N van Mechelen W Straker LM. Do highly physically active workers die early? A systematic review with meta-analysis of data from 193 696 participants. Br J Sports Med. (2018) 52:1320–6. 10.1136/bjsports-2017-098540 29760168

[39] Hallman DM Mathiassen SE Gupta N Korshøj M Holtermann A. Differences between work and leisure in temporal patterns of objectively measured physical activity among blue-collar workers. BMC Public Health. (2015) 15:976. 10.1186/s12889-015-2339-4 26415931 PMC4587719

[40] Jung HS Chang Y Kwon MJ Sung E Yun KE Cho YK. Smoking and the risk of non-alcoholic fatty liver disease: a cohort study. Am J Gastroenterol. (2019) 114:453–63. 10.1038/s41395-018-0283-5 30353055

[41] Sakuma T Nakamura M Chiba T Iwanaga T Kan M Kojima R. A diet-induced murine model for non-alcoholic fatty liver disease with obesity and insulin resistance that rapidly develops steatohepatitis and fibrosis. Lab Invest. (2022) 102:1150–7. 10.1038/s41374-022-00807-6 35643859

[42] Scorletti E Carr RM. A new perspective on NAFLD: focusing on lipid droplets. J Hepatol. (2022) 76:934–45. 10.1016/j.jhep.2021.11.009 34793866

[43] Rinella ME. Nonalcoholic fatty liver disease: a systematic review. JAMA. (2015) 313:2263–73. 10.1001/jama.2015.5370 26057287

[44] Holtermann A Schnohr P Nordestgaard BG Marott JL. The physical activity paradox in cardiovascular disease and all-cause mortality: the contemporary Copenhagen General Population Study with 104 046 adults. Eur Heart J. (2021) 42:1499–511. 10.1093/eurheartj/ehab087 33831954 PMC8046503

[45] Meyfroidt G Baguley IJ Menon DK. Paroxysmal sympathetic hyperactivity: the storm after acute brain injury. Lancet Neurol. (2017) 16:721–9. 10.1016/S1474-4422(17)30259-4 28816118

[46] Öhlin J Liv P Andersson M Järvholm B Slunga Järvholm L Stjernbrandt A. Occupational physical activity and resting blood pressure in male construction workers. Int Arch Occup Environ Health. (2023) 96:1283–9. 10.1007/s00420-023-02006-2 37725195 PMC10560137

[47] Wood AM Kaptoge S Butterworth AS Willeit P Warnakula S Bolton T. Risk thresholds for alcohol consumption: combined analysis of individual-participant data for 599 912 current drinkers in 83 prospective studies. Lancet. (2018) 391:1513–23. 10.1016/S0140-6736(18)30134-X 29676281 PMC5899998

[48] Di Federico S Filippini T Whelton PK Cecchini M Iamandii I Boriani G. Alcohol intake and blood pressure levels: a dose-response meta-analysis of nonexperimental cohort studies. Hypertension. (2023) 80:1961–9. 10.1161/HYPERTENSIONAHA.123.21224 37522179 PMC10510850

[49] Vacca A Bulfone L Cicco S Brosolo G Da Porto A Soardo G. Alcohol intake and arterial hypertension: retelling of a multifaceted story. Nutrients. (2023) 15:958. 10.3390/nu15040958 36839317 PMC9963590

[50] Long MT Zhang X Xu H Liu CT Corey KE Chung RT. Hepatic fibrosis associates with multiple cardiometabolic disease risk factors: the Framingham Heart Study. Hepatology. (2021) 73:548–59. 10.1002/hep.31608 33125745 PMC8515503

[51] Xiong S Yin S Deng W Zhao Y Li W Wang P. Predictive value of liver fibrosis scores in cardiovascular diseases among hypertensive population. J Hypertens. (2023) 41:741–50. 10.1097/HJH.0000000000003394 36883472 PMC10090336

[52] Stahl EP Dhindsa DS Lee SK Sandesara PB Chalasani NP Sperling LS. Nonalcoholic fatty liver disease and the heart: JACC state-of-the-art review. J Am Coll Cardiol. (2019) 73:948–63. 10.1016/j.jacc.2018.11.050 30819364

[53] Targher G Byrne CD Lonardo A Zoppini G Barbui C. Non-alcoholic fatty liver disease and risk of incident cardiovascular disease: a meta-analysis. J Hepatol. (2016) 65:589–600. 10.1016/j.jhep.2016.05.013 27212244

[54] Gao J Wei Q Pan R Yi W Xu Z Duan J. Elevated environmental PM(25) increases risk of schizophrenia relapse: mediation of inflammatory cytokines. Sci Total Environ. (2021) 753:142008. 10.1016/j.scitotenv.2020.142008 32892002

[55] Sakboonyarat B Poovieng J Lertsakulbunlue S Jongcherdchootrakul K Srisawat P Mungthin M. Association between raised blood pressure and elevated serum liver enzymes among active-duty Royal Thai Army personnel in Thailand. BMC Cardiovasc Disord. (2023) 23:143. 10.1186/s12872-023-03181-3 36944947 PMC10029162

[56] Pang Y Lv J Kartsonaki C Yu C Guo Y Chen Y. Genetic and healthy lifestyle factors in relation to the incidence and prognosis of severe liver disease in the Chinese population. Chin Med J. (2023) 136:1929–36. 10.1097/CM9.0000000000002754 37476847 PMC10431401

[57] Zelber-Sagi S Noureddin M Shibolet O. Lifestyle and hepatocellular carcinoma what is the evidence and prevention recommendations. Cancers. (2021) 14:103. 10.3390/cancers14010103 35008267 PMC8750465

[58] He H Zhang T Zhou J Zhu Z Na X Zhou G. Associations of physical activity and egg intake with hypertension among Chinese middle-aged and older population. Sci Rep. (2019) 9:7722. 10.1038/s41598-019-43966-1 31118447 PMC6531484

