# Supplementary material for: Lifestyle factors modified the mediation role of liver fibrosis in the association between occupational physical activity and blood pressure
Source: Front Public Health. 2024 Jun 26;12:1383065. doi: 10.3389/fpubh.2024.1383065 (PMC11233708; doi:10.3389/fpubh.2024.1383065)
Supplement: Supplementary file 1 [file Data_Sheet_1.docx]

Supplementary Material

Lifestyle factors modified the mediation role of liver fibrosis in the association between occupational physical activity and blood pressure

Shangyi Zhang1,2†, Zhenlong Chen1†, Xinman Jiang1†, Shenglan Zhou1, Yanru Liu1, Mingsheng Liu1,2, Xiayun Dai1, Bifeng Lu1, Guilin Yi1, Wenjun Yin1,2*

*** Correspondence:**Prof. Wenjun Yin, Wuhan Prevention and Treatment Center for Occupational Diseases, Jianghan Bei Lu 18, Wuhan, 430015, Hubei, PR China. Email:ywjyjs@foxmail.com.

# Supplementary Figures and Tables

## Supplementary Figures


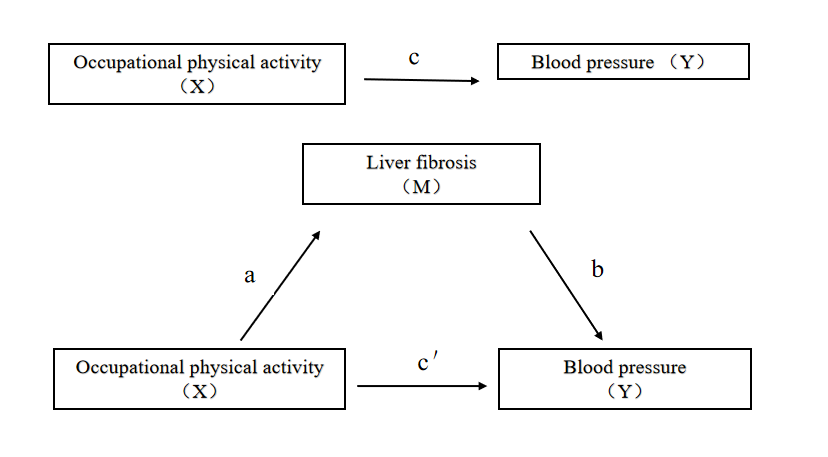


**Supplementary Figure 1.** Mediation effect analysis. c is the total effect of X on Y, a × b is the mediating effect through the mediating variable M, c' is the direct effect. The total effect = direct effect + mediated effect (c = c' + a × b).


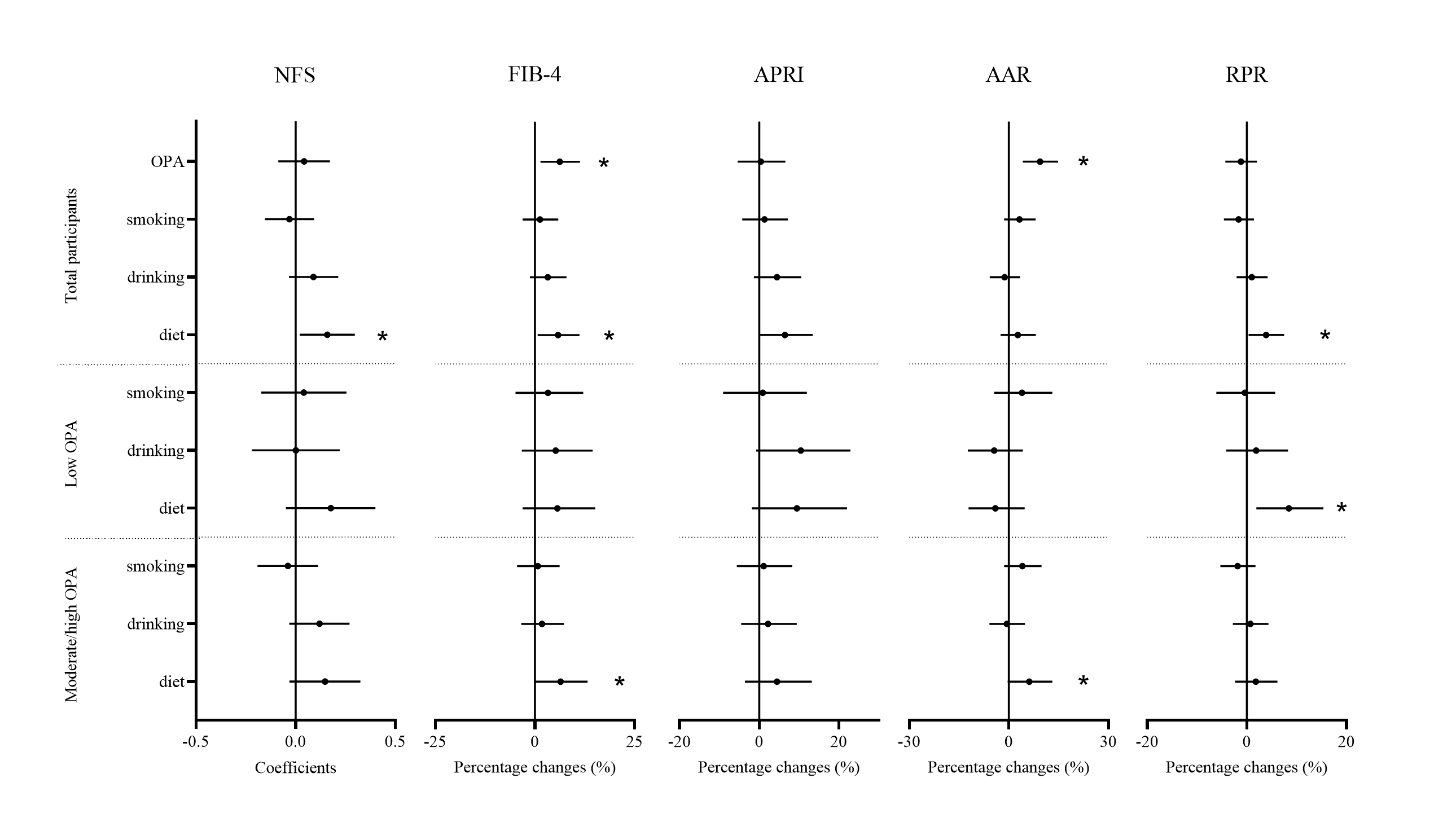


**Supplementary Figure 2.** Association of occupational physical activity and Lifestyle factor with the liver fibrosis indices. Result from generalized additive model adjusted for age, sex, body mass index, job seniority, married, average monthly earnings. The results represented estimated changes and 95% confidence intervals for association between liver fibrosis indices, OPA, and Lifestyle factor. Abbreviations: OPA: occupational physical activity; drinking: drinking alcohol; diet:diet habits; NFS: non-alcoholic fatty liver disease fibrosis score; FIB-4: fibrosis index based on the four factors; APRI: aspartate aminotransferase to platelet ratio index; AAR: aspartate aminotransferase to alanine aminotransferase ratio; RPR: red blood cell distribution width to platelet. **P* < 0.05

## Supplementary Tables

| **Supplementary Table 1.** Mediating effect of liver fibrosis on the association of OPA with BP | | | | | | |
| --- | --- | --- | --- | --- | --- | --- |
| Parameters |  | Exposure to mediator  (β_exposure_) | Mediator to outcome  (γ_M_) | Mediated effect  (Indirect effect, β_exposure_×γ_M_) | Direct effect  (γ_exposure_) | Mediated proportion (%) |
| SBP | NFS | 0.44 (0.29, 0.59) ^*^ | 2.86 (1.92, 3.81) ^*^ | 1.27 (0.74, 1.95) ^*^ | 4.36 (2.04, 6.69) ^*^ | 22.56% |
|  | FIB-4 | 0.27 (0.21, 0.33) ^*^ | 8.56 (6.28, 10.83) ^*^ | 2.33 (1.60, 3.27) ^*^ | 3.30 (0.95, 5.65) ^*^ | 41.39% |
|  | APRI | 0.04 (-0.01, 0.10) | 2.64 (-0.0004, 5.28) | 0.11 (-0.01, 0.42) | 5.52 (3.19, 7.84) ^*^ | - |
|  | AAR | 0.13 (0.08, 0.17) ^*^ | 0.29 (-2.98, 3.55) | 0.04 (-0.38, 0.44) | 5.59 (3.23, 7.96) ^*^ | - |
|  | RPR | 0.02 (-0.01, 0.05) | 1.87 (-2.91, 6.66) | 0.04 (-0.05, 0.29) | 5.59 (3.26, 7.92) ^*^ | - |
| DBP | NFS | 0.44 (0.29, 0.59) ^*^ | 1.13 (0.51, 1.75) ^*^ | 0.50 (0.23, 0.90) ^*^ | 1.96 (0.44, 3.48) ^*^ | 20.33% |
|  | FIB-4 | 0.27 (0.21, 0.33) ^*^ | 3.67 (2.18, 5.17) ^*^ | 1.00 (0.55, 1.54) | 1.46 (-0.08, 3.00) | - |
|  | APRI | 0.04 (-0.01, 0.10) | 0.96 (-0.75, 2.67) | 0.04 (-0.02, 0.21) | 2.42 (0.92, 3.93) ^*^ | - |
|  | AAR | 0.13 (0.08, 0.17) ^*^ | -0.47 (-2.58, 1.64) | -0.06 (-0.37, 0.21) | 2.52 (0.99, 4.05) ^*^ | - |
|  | RPR | 0.02 (-0.01, 0.05) | 0.62 (-2.47, 3.72) | 0.01 (-0.05, 0.16) | 2.45 (0.94, 3.96) ^*^ |  |
| HR | NFS | 0.44 (0.29, 0.59) ^*^ | -1.03 (-1.64, -0.43) ^*^ | -0.46 (-0.87, -0.16) | -1.45 (-2.94, 0.04) | - |
|  | FIB-4 | 0.27 (0.21, 0.33) ^*^ | -3.60 (-5.07, -2.14) ^*^ | -0.98 (-1.58, -0.54) | -0.93 (-2.44, 0.58) | - |
|  | APRI | 0.04 (-0.01, 0.10) | -2.31 (-3.98, -0.64) ^*^ | -0.10 (-0.31, 0.01) | -1.81 (-3.28, -0.34) ^*^ | - |
|  | AAR | 0.13 (0.08, 0.17) ^*^ | -3.72 (-5.77, -1.66) ^*^ | -0.48 (-0.87, -0.21) | -1.43 (-2.92, 0.06) | - |
|  | RPR | 0.02 (-0.01, 0.05) | -6.50 (-9.50, -3.49) ^*^ | -0.13 (-0.41, 0.05) | -1.77 (-3.24, -0.31) ^*^ | - |
| Hypertension | NFS | 0.44 (0.29, 0.59)^*^ | 0.34 (0.20, 0.48)^*^ | 0.15 (0.08, 0.25) | 0.24 (-0.10, 0.59) | - |
|  | FIB-4 | 0.27 (0.21, 0.33)^*^ | 1.02 (0.68, 1.36)^*^ | 0.28 (0.18,0.50) | 0.14 (-0.21, 0.49) | - |
|  | APRI | 0.04 (-0.01, 0.10) | 0.32 (-0.03, 0.67) | 0.01 (-0.00, 0.05) | 0.36 (0.03, 0.70)^*^ | - |
|  | AAR | 0.13 (0.08, 0.17) ^*^ | -0.09 (-0.55, 0.36) | -0.01 (-0.07, 0.05) | 0.39 (0.05, 0.73)^*^ | - |
|  | RPR | 0.02 (-0.01, 0.05) | 0.71 (0.06, 1.35)^*^ | 0.015(-0.00, 0.06) | 0.36 (0.03, 0.70)^*^ | - |
| Pre-hypertension | NFS | 0.44 (0.29, 0.59)^*^ | 0.03 (-0.08, 0.14) | 0.01 (-0.04, 0.07) | 0.35 (0.08, 0.63)^*^ | - |
|  | FIB-4 | 0.27 (0.21, 0.33)^*^ | 0.12 (-0.15, 0.39) | 0.03 (-0.04, 0.11) | 0.33 (-0.05, 0.61) | - |
|  | APRI | 0.04 (-0.01, 0.10) | -0.01 (-0.32, 0.29) | -0.001 (-0.02,0.01) | 0.37 (0.10, 0.64) | - |
|  | AAR | 0.13 (0.08, 0.17) ^*^ | 0.06 (-0.32, 0.43) | 0.01 (-0.04, 0.06) | 0.36 (0.09, 0.63) | - |
|  | RPR | 0.02 (-0.01, 0.05) | -0.46 (-1.02, 0.10) | -0.01 (-0.04, 0.003) | 0.38 (0.11, 0.65) | - |
| OPA: occupational physical activity; drinking: drinking alcohol; diet:diet habits; NFS: non-alcoholic fatty liver disease fibrosis score; FIB-4: fibrosis index based on the four factors; APRI: aspartate aminotransferase to platelet ratio index; AAR: aspartate aminotransferase to alanine aminotransferase ratio; RPR: red blood cell distribution width to platelet. ^*^*P* < 0.05. | | | | | | |
